# A fatal case of traumatic brain injury with severe coagulopathy due to *Rhabdophis tigrinus* (yamakagashi) bites: a case report

**DOI:** 10.1186/s12245-021-00402-4

**Published:** 2021-12-20

**Authors:** Daisuke Ueno, Shiino Yasukazu, Jiro Takahashi, Satomi Miyamoto, Takahiro Inoue

**Affiliations:** Department of Acute Medicine, Kawasaki Medical School, 577 Matsushima, Kurashiki, Okayama, 701-0192 Japan

**Keywords:** Recombinant thrombomodulin, Disseminated with a fibrinolytic phenotype, Antivenom

## Abstract

**Background:**

Yamakagashi venom is a prothrombin activator, leading to disseminated intravascular coagulation. We report a fatal case of severe coagulopathy from head trauma assumed to be caused by a yamakagashi bite.

**Case presentation:**

An 80-year-old man fell and developed systemic tonic–clonic convulsions. Head computed tomography revealed brain contusion and acute subdural hematoma. Physical examination revealed two bite marks with persistent bleeding on the right lower leg. The patient stated that he had been bitten by some creature 3 days prior, but the bite was left untreated. Laboratory tests showed fibrinogen levels below the detection limit. Although eighteen units of fresh frozen plasma were administered for coagulopathy, fibrinogen levels did not improve. He died about 18 h after a head injury.

**Conclusion:**

In this case of a yamakagashi bite with active bleeding due to trauma, early administration of yamakagashi antivenom should be considered to control coagulopathy.

## Background

Although the exact number is unknown, there have been thirty-seven cases of 40 years, making these instances relatively rare [1]. Only one fatal case has been reported since 2000 due to the early administration of yamakagashi antivenom in severe cases. Here, we report a fatal case of severe coagulopathy from head trauma assumed to be caused by a yamakagashi bite.

## Case presentation

**Fig. 1:**
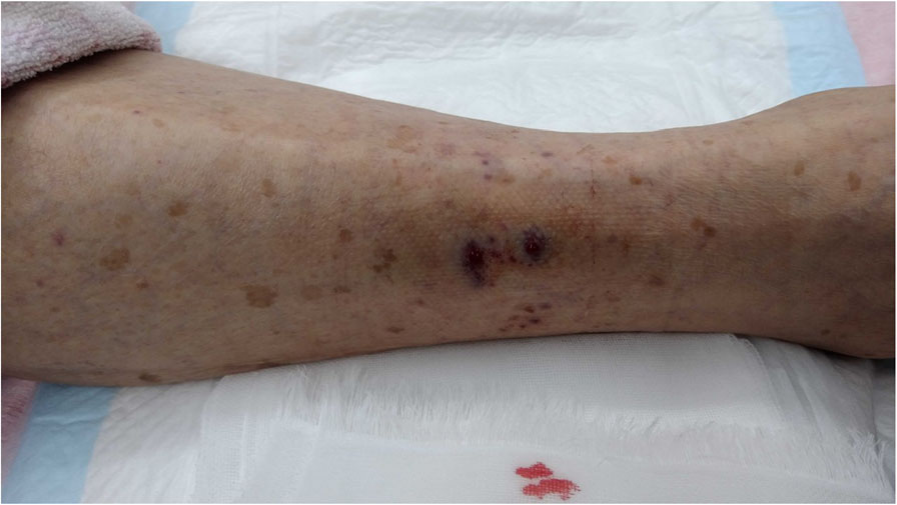
The patient’s right lower leg. Two bite wounds of a few millimeters on the patient’s right lower leg are observed. The wound showed persistent bleeding and swelling even though it was several days before the injury

**Fig. 2:**
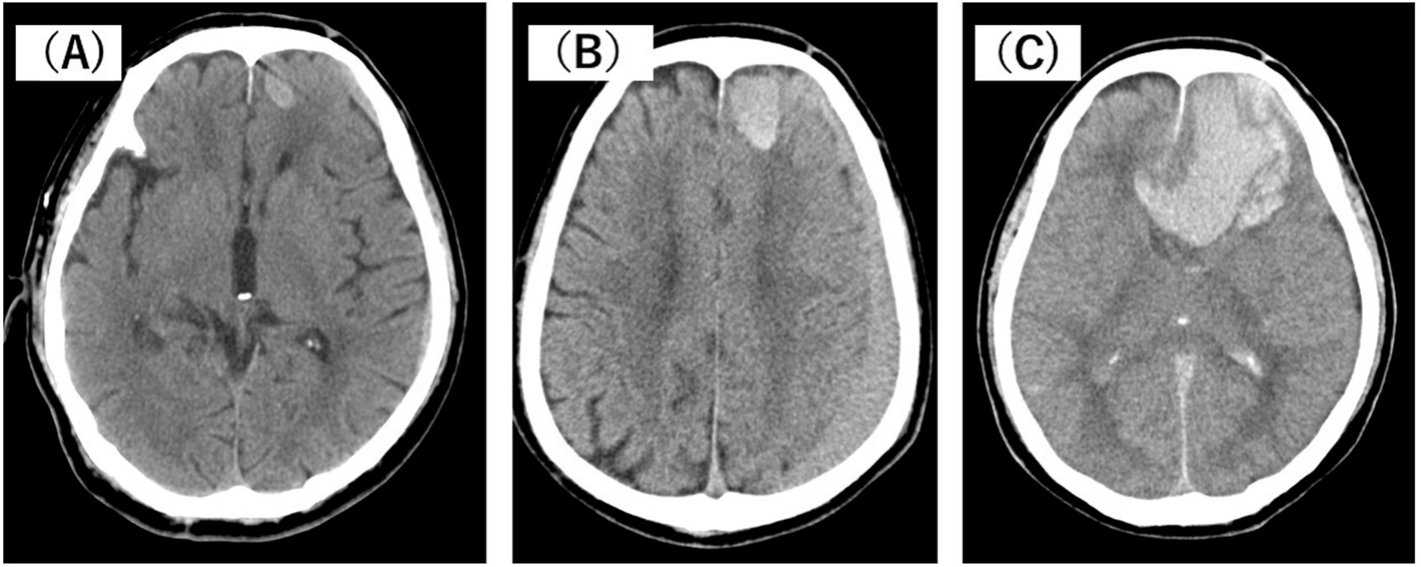
The clinical course of intracranial lesions in the head computed tomography (CT). **A** Head CT immediately after the head injury showed cerebral contusion in the left frontal lobe and a left subacute subdural hematoma (performed at a previous hospital). **B** Two hours after the head injury, the left subacute subdural hematoma was not significantly changed, but the brain contusion worsened. **C** Seven hours after the head injury, the hematoma in the left frontal lobe was markedly enlarged and had perforated into the ventricle

**Fig. 3:**
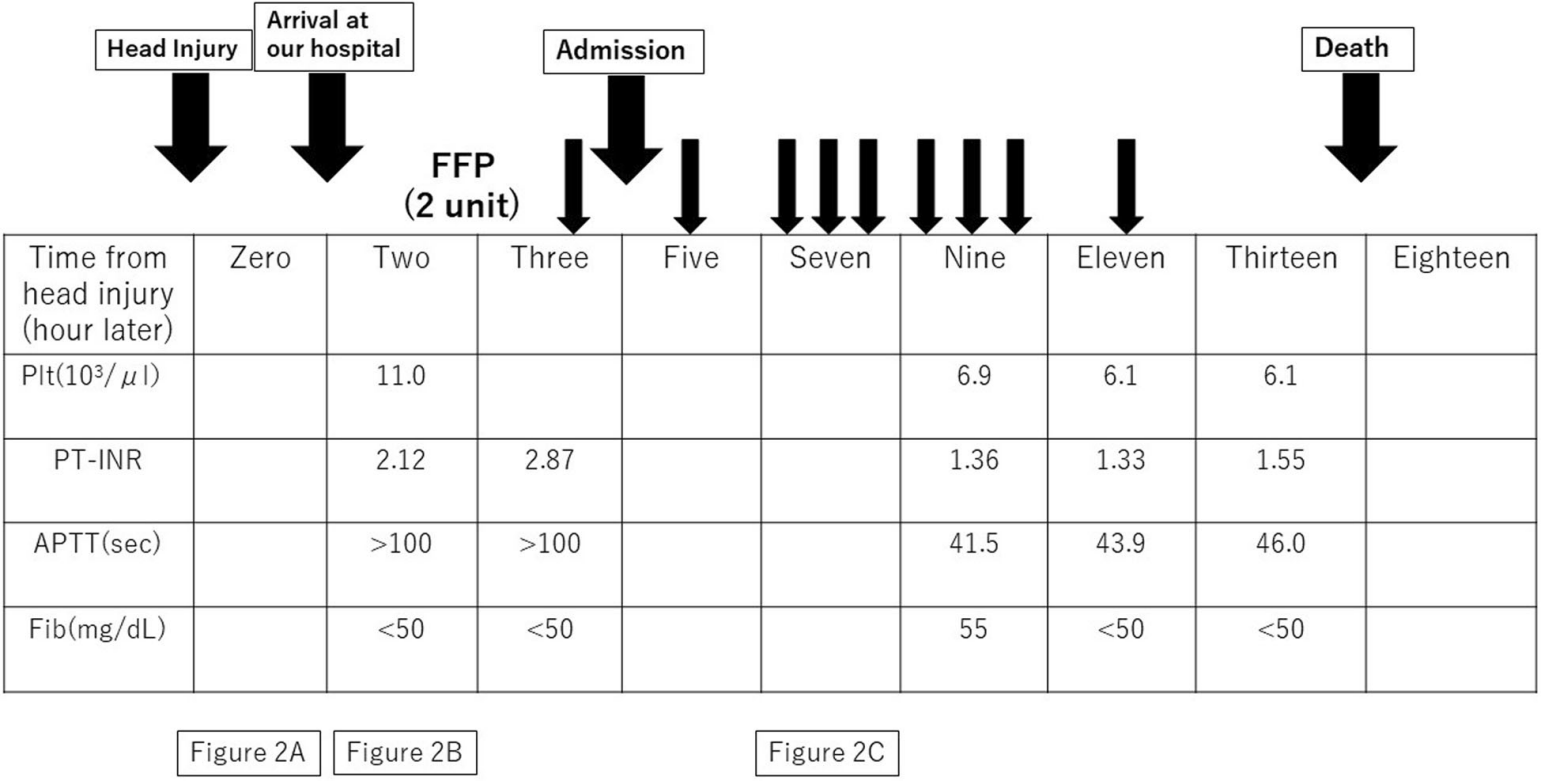
The clinical course of coagulopathy and head CT. FFP, fresh frozen plasma; Plt, platelet; PT-INR, prothrombin time-international normalized ratio; APTT, activated partial thromboplastin time; Fib, fibrinogen. When FFP administration was started, PT-INR and APTT showed a transient improvement, but Fib remained almost below the detection limit

An 80-year-old man visited a neighboring hospital complaining of back pain. He accidentally fell in the waiting room and subsequently developed systemic tonic–clonic convulsions. The patient was subsequently transferred to our hospital. His vital signs appeared normal; however, a mild disturbance of consciousness was observed (Glasgow Coma Scale E4V4M6). The light reflex was prompt on both sides, and there was no anisocoria. Physical examination revealed two swollen and continuously bleeding bite marks on the right lower leg (Fig. 1). The patient stated that he had been bitten by a creature 3 days prior and left his bite untreated. No other physical or trauma-related changes were observed, and no abnormalities except coagulopathy were noted on laboratory tests (Table 1). The patient showed a prolonged prothrombin and activated partial thromboplastin time, and fibrinogen levels were below the detection limit. Head computed tomography (CT) performed at the previous hospital revealed a single cerebral contusion in the left frontal lobe and a left subacute subdural hematoma (Fig. 2A). Follow-up head CT performed 2 h after the head injury revealed a single brain contusion and a small amount of acute subdural hematoma in the left frontal lobe (Fig. 2B). Accordingly, the patient was diagnosed with a head injury accompanied by severe coagulopathy and admitted to the intensive care unit. The neurosurgeon found no indications for surgery. Head CT performed 7 h after the head injury revealed gradual worsening of intracranial hemorrhage (Fig. 2C). For coagulopathy, eighteen units of fresh frozen plasma (FFP) was administered about 8 h after arrival at our hospital, but fibrinogen levels showed little improvement (Fig. 3). Furthermore, his state of consciousness deteriorated over time. However, surgical intervention was not indicated because of severe coagulopathy. The patient died 3 days after the bite, about 18 h after head injury.

**Table 1:** Laboratory examination results on arrival at our hospital

WBC	6840	/μL	TP	6.6	g/dL	Na	142	mEq/L
Neut	78.4	%	Glu	132	mg/dL	K	3.9	mEq/L
Mono	5.8	%	T-Bil	1.5	mg/dL	Cl	109	mEq/L
Eos	0.3	%	ALP	77	IU/L			
Lym	15.1	%	T-cho	197	mg/dL	BGA (room air)		
Baso	0.4	%	γ-GTP	26	IU/L	pH	7.404	
RBC	442 × 10^4^	/μL	LDH	245	IU/L	PaO2	84.7	mmHg
Hb	13.9	g/dL	Alb	4.3	g/dL	PaCO2	38.0	mmHg
Ht	39.2	%	Glb	2.3	g/dL	BE	− 1.2	mmol/L
Plt	11.0 × 10^4^	/μL	ChE	256	IU/L	Lac	1.14	mmol/L
			AST	21	IU/L			
PT-INR	2.12		ALT	14	IU/L			
APTT	> 100	sec	Cr	0.64	mg/L			
Fib	< 50	mg/dL	BUN	30	mg/dL			
d-dimer	112.0	μg/mL	CRP	0.10	mg/dL			

*WBC*, white blood cell; *Neut*, neutrophil; *Mono*, monocyte; *Eos*, eosinophil; *Lym*, lymphocyte; *Baso*, basophil; *RBC*, red blood cells; *Hb*, hemoglobin; *Ht*, hematocrit; *Plt*, platelet; *PT-INR*, prothrombin time-international normalized ratio; *APTT*, activated partial thromboplastin time; *Fib*, fibrinogen; *TP*, total protein; *Glu*, glucose; *T-Bil*, total bilirubin; *ALP*, alkaline phosphatase; *T-cho*, total cholesterol; *γ-GTP*, gamma glutamyl transpeptidase; *LDH*, lactate dehydrogenase; *Alb*, albumin; *Glb*, globulin; *ChE*, cholinesterase; *AST*, aspartate aminotransferase; *ALT*, alanine aminotransferase; *Cr*, creatinine; *BUN*, blood urea nitrogen; *CRP*, C-reactive protein; *Na*, sodium; *K*, potassium; *Cl*, chloride; *BGA*, blood gas analysis; *PaO*_*2*_, partial pressure of arterial oxygen; *PaCO*_*2*_, partial pressure of arterial carbon dioxide; *BE*, base excess; *Lac*, lactate

## Discussion

In this case, a definite diagnosis of a yamakagashi bite could not be made. However, the patient had two bite wounds on the right lower leg, the bleeding persisted 3 days after the injury, coagulopathy with fibrinogen levels below fifty mg/dL was present, and fibrinogen levels remained below detectable levels despite FFP administration. Therefore, the severe head injury caused by coagulopathy likely resulted from a yamakagashi bite.

Yamakagashi is found throughout Russia and eastern Asia, including China, Taiwan, Korea, and Japan (excluding the Ryukyu region), and they produce two kinds of venom [2, 3]. Cervical gland venom is released from about ten pairs of glands under the skin of the neck when the glands rupture as the neck is strongly compressed, and it causes corneal ulceration and iritis when it enters the eye. Duvernoy’s gland venom, in contrast, is released from the hind region of the maxilla through conduit openings in front of the two pairs of fangs located on the back teeth of the maxilla. A momentary bite is often not enough to release the venom because these fangs are short (< 2 mm), and no muscles are needed to squeeze the Duvernoy’s gland. If the back teeth are involved in the bite, this venom can enter the body and cause serious bleeding.

Yamakagashi venom is a prothrombin activator. It causes strong blood coagulation and has a weak thrombin-like effect [4], leading to disseminated intravascular coagulation (DIC) with a fibrinolytic phenotype [5]. Disseminated fibrin formation occurs, and fibrinolysis is activated, causing hypofibrinogenemia and increased levels of fibrinogen degradation products [1].

Therapies for DIC due to yamakagashi venom include serotherapy (definitive therapy) and recombinant thrombomodulin (rTM) therapy (alternative therapy). Yamakagashi antivenom can only be administered in clinical trials. Although yamakagashi antivenom has an equine source, anaphylaxis rates are 0% [6]. In one study, compared to the antivenom non-administered group (*n* = 15), the antivenom administered group (*n* = 19) had a better prognosis (*p* = 0.03) and lower incidence of renal failure requiring dialysis (*p* = 0.03) [1]. Since yamakagashi antivenom is used off-label in Japan [2], providers must participate in a clinical research group in order to administer it [5].

Additionally, considering that yamakagashi venom causes DIC with a fibrinolytic phenotype, rTM preparations are expected to be effective [7], and their applications are being investigated. rTM can inhibit thrombin production, thus reducing bleeding symptoms and organ damage caused by DIC [8]. However, rTM is contraindicated in early phases of trauma because it may promote bleeding, and the patient described in this report exhibited an active intracranial hemorrhage. To our knowledge, there have been no reported cases of complications in cases of traumatic intracranial hemorrhage after yamakagashi bites due to rTM administration. Moreover, only one report of rTM use in the diagnosis of DIC due to trauma exists, and the efficacy of rTM has not been reported [9]—though no life-threatening bleeding events have been observed in patients who die. Yamakagashi bites can cause DIC with a fibrinolytic phenotype; however, rTM may not be used in cases of yamakagashi bites with active bleeding due to trauma.

In this case, the possibility of a yamakagashi bite had not been considered, and a large dose of FFP was administered to improve trauma-related severe coagulopathy. However, the intracranial hematoma worsened, with little improvement in fibrinogen levels, causing death.

Due to the active bleeding caused by trauma, even upon a diagnosis of the yamakagashi bite, there would have been no definitive treatment for the injury aside from yamakagashi antivenom.

## Conclusion

In this case of a yamakagashi bite coupled with active bleeding due to trauma, yamakagshi antivenom would have been the only definitive treatment option for the patient.

## Data Availability

The datasets used and/or analyzed during the current study are available from the corresponding author on reasonable request.

## Abbreviations

CT: Computed tomography
FFP: Fresh frozen plasma
DIC: Disseminated intravascular coagulation
rTM: Recombinant thrombomodulin
